# What Is the Physician’s Duty in the Prevention of Moral and Social Diseases?1President’s address delivered during the Forty-second annual meeting of the Central New York Medical Association held at Syracuse, October 19 and 20, 1910.

**Published:** 1911-02

**Authors:** F. W. Sears

**Affiliations:** Syracuse, N. Y. 709 South Warren Street


### What is the Physician's Duty in the Prevention of Moral and Social Diseases? [1]

By F. W. SEARS. M.D.
Syracuse, N. Y.

"A child's education should begin one hundred years before its birth."
—*Emerson*

ONE cannot thoughtfully study the great social ills of today without being convinced that the moral training of a child should begin at least a generation before it is born. In my decision to present such an unpleasant subject before you today, I do so with the hope that I may enlist your thoughtful consideration of a problem, in the solution of which, physicians must assume a very important role. We are in a position to observe the results of the present tendencies to disregard the fundamental principles of life.

Many will point with pride to their ancestors, and yet will not take the slightest interest in the fact that they should become ancestors of the future generations. Selfishness and self-indulgence is the worm that is at the roots and branches of the great social tree, and the man or woman who, from selfish motives shrinks from the obligations of parenthood, is assisting in destroying the life and beauty of that tree.

The increasing prevalence of criminal abortion is an evil, which is undermining the very foundation of our social structure. Re-

1. President's address delivered during the Forty-second annual meeting of the Central New York Medical Association held at Syracuse, October 19 and 20, 1910.

production is the most sacred duty with which we are intrusted, yet how many enter the marriage state with this thought in their minds? The present tendency is to shirk the responsibility and self-sacrifice necessary to the rearing of children, one of the most fruitful causes of prostitution. Deliberate plans are laid to prevent conception, and if, by chance, it does take place what proportion of the innocent lives are doomed to destruction?

Dr. C. S. Bacon, states that from 20% to 25% of all pregnancies end in abortion. (1) The conclusions of a committee appointed by the Board of Health of Michigan were, that one-third of all pregnancies end in abortion, and that 6,000 women die annually, in the United States from this cause. (2) A public official estimates that not less than 100,000 abortions are committed in New York state every year, 10,000 in the city of Chicago annually; (3) and one of the most significant facts is, that those who are best fitted physically, and by education, to rear families are the most proficient in avoiding that responsibility, as is indicated by the fact that 27% of all college educated women are barren. (4) Eugenic laws may do much, but how can we correct these conditions? (5) Much of it is the result of ignorance. It is quite generally believed by the laity, and has been so held by the laws of many states, that the fetus is not a separate individual until quickening has taken place, but many of the more recent laws do not recognise any such distinction.

I believe it is the duty of the physician to dispel these absurd opinions. We know that individual life begins with conception. Life begins when that tiny fertilised ovum reaches its nest in the uterus. It is not then a part of the maternal tissues to be discarded at her discretion. It is endowed with a separate life, a soul, an individuality. The mother's relations to it are simply those of nutrition and protection. She holds the same relation to it, that she does to the child at her breast, and the one who seeks to destroy this life is equally guilty whether he or she does it ten days after conception or ten days before full gestation. I doubt if we could find a woman so hardened in crime, that she would come into a physician's office and ask him to kill her husband, because he was an inconvenience to her; yet the only reason that it is a greater crime is because of the benumbed and lax public sentiment which has so long tolerated the crime of feticide. Who are the abortionists? I doubt if there are many physicians today, who could be induced to perform criminal abortion. Very many of them are self-induced, and many are caused by midwives, and at the direction of a would-be friend in need. And what are the results? Invalidism and a lowering of moral standards, filling our hospitals and divorce courts.

I doubt if a woman ever perfectly regains her normal condition after an induced abortion. It is crime which nature keenly resents. A learned judge of Detroit states that the majority of women, who apply for divorce, are childless. Professor M. Claire of Paris states, that one-third of the patients in his surgical ward are cases of the febrile sequellæ of criminal abortion, and generally self-induced abortion. (6) I believe that most hospitals would equal that proportion caused by pelvic abscess and serious inflammations and hemorrhages of the woman, many of them resulting in loss of life from serious infection, and should she escape the more serious physical results, she cannot escape the moral influence of crimes of this nature. One cannot teach high moral principles successfully, unless he practices morality and the woman, who tolerates or practices abortion upon herself, thereby lowers her moral tone, and weakens the marriage relations and her home influences to such an extent that she renders herself unfit to train her sons and daughters to become noble men and women. It is in the early moral training and careful education in matters pertaining to sex that we must turn our attention, if we can hope to, in the slightest degree, mitigate the evil of prostitution and its resultant great black plague of venereal diseases, which in its effects far exceeds, by its morbidity and mortality, the great white plague, which is justly receiving so much attention at present, because it pollutes the very fountain springs of life.

Venereal diseases existed centuries before the Christian Era, and will probably exist for centuries to come: as Dr. Kelly expressed it, "It is like bubbles on the surface of a seething caldron, you may skim them off but more keep rising to the top." Because we cannot control it is no reason why we should not individually do a great deal. (7) And it is for this that societies for moral and social prophylactics are being formed in most of the larger cities.

Reliable statistics of venereal diseases are difficult or quite impossible to obtain. Boards of health do not include them among the diseases to be reported. Hospital boards do not like to have them appear on their records, for fear their name would contaminate their hospital, and the individual makes every effort to have his malady kept a secret. This secrecy is one of the chief obstacles in the way of better conditions. Estimates made by competent physicians from the most reliable data, show that venereal diseases far outnumber all other contagious diseases. In 1900 the number of cases reported to the Board of Health of New York City of tuberculosis, scarlatina, measles, and diphtheria combined was 41,145, while it was estimated that there were, during that year, in New York City, 225,000 cases of venereal diseases. (8) Letters to physicians in New York City indicated that there were treated in private practice in 1901, 162,372 cases of venereal disease. (9) It is claimed that 80% of the men in New York City, either have or have had gonorrhea, and that probably 90% of them have not been cured, and that at least 10% of the men have had syphilis. (10) Noeggarth states that in every 1,000 married men in New York 800 have had gonorrhea. (11) Hoff estimates that there are in the United States today at least one-half million of people affected with syphilis. (12) From these estimates Rev. C. D. Case states that of the 770,000 young men who attain the age of puberity every year, 450,000 will become affected with venereal disease. (13) And what are the results? 450,000 foci of infection at large, spreading its poison among thousands of innocent victims, carrying diseases and death in their wake, ignorant, many of them, of the channels through which infection may be carried, often indifferently treated, with no one to raise a protest except the feeble voice of his starved and sickly conscience, and you and I must continue to witness some of these young men becoming married to pure and beautiful young women, who little suspect that they may be obliged to endure a life of suffering, a childless home, or perhaps worse, and our lips are sealed by professional secrecy!

Must we always be thus bound? Are we not carrying professional secrecy farther than we have either a moral or legal right to do? Is it not cowardly in us to thus allow women and children to become the innocent victims of this terrible scourge and to assume the burden of the results of men's debauchery? And we acquiesce by our silence. Lawyer Purrington of New York, says, "If a client comes to a lawyer and says 'I have committed a crime, defend me," the secret is kept, but if he comes saying 'I intend to commit a certain crime,' it is the lawyer's duty to prevent him." Why should not we take the same view when a young man with venereal disease disregards our advice not to marry until cured? Should we not do all in our power to prevent it? Let us look at some of the results of such marriages. One-third of all gonorrhea in women is acquired from their husbands; 50% of women who have had gonorrhea are sterile; 75% of all operations performed in hospitals on women are the results of gonorrhea disease. Dr. Joseph Price states that of 1,000 operations performed on women for pelvic disease, 95% were the result of gonorrhea.

There were in 1900, 101,123 blind people in the United States, over 20,000 of which were caused by gonorrhea, as 20% of all blindness is caused by this disease. Woods Hutchinson states that 50% of the children of syphilitic parents are either still born or die during the first six months. (14) Fournier says 77% of all children with inherited syphilis die. In a record of 43 syphilitic marriages, with 206 pregnancies, 162 children were born alive, 81 had meningitis and convulsions; and brain affection occurred 12 times. (15) In three syphilitic couples 22 children were born, only one of which grew up to healthy adult life. (16) Zicheu states that 10% of idiots have inherited syphilis. Is this not an appalling harvest of innocent suffering? And this does not include the thousands who suffer in silence, martyrs to their pride and the honor of their families. The great foul stream which spreads this pollution into every country and every city, has but one name the world over, prostitution. States and municipalities have tried in vain to check its effects by trying to purify its polluted streams, while they have not paid the slightest attention to the sources from which they spring. Remove every prostitute from the country, today, and within six months the place of every one will be filled. So long as there is a demand, there will be a supply. You may have perfect laws, and you may have them faultlessly enforced, but you cannot raise the standard of your community to a higher plane than the average of the individual moral character of your citizens. Judge Corrigan says "Laws cannot change the nature of man." Licensing and regulation of prostitutes have been a failure the world over. Most extensive systems of examination, regulation and restriction exist in Berlin, in Paris and other European cities, but in only one (Dresden) which has the most thorough system of examination, has there been any diminution in the amount of venereal diseases, and not in one has prostitution decreased.

In some cities the number of acknowledged prostitutes has been diminished, but the clandestined have increased to a far greater extent. One might as well cover the chimney which emits its foul smoke and pay no attention to the furnace below. You might thus diminish the visible foulness, only to force it through the crevices into the very foundation of your homes. You cannot have regulation without recognising its right to exist. Its existence according to our laws is a crime. If it has a right to exist it has a right to increase its membership. If it has a right to increase its membership, from what ranks in life shall we enlist them? The life of a prostitute is from 6 to 9 years, and their death rate is 75 per cent. greater than the normal woman. The present system of police regulation in our own cities is in effect but a very poor system of licensing. Only in rare instances does the sentence amount to more than a fine, which only stimulates the inmate to renewed efforts to make up the loss sustained. In 1907 in one district in New York City 367 cases were made against 115 places with but two prison sentences given and $10,000.00 in fines collected. (27) The political official has his finger ever on the pulse of public sentiment and raids are timed according to the symptoms, and are always spasmodic, but seldom reach the worst offenders. I believe the time will come when health authorities will be obliged to meet this problem in a practical way the same as they do in all other contagious diseases. There is today no law in any country which allows restriction to be placed on a man suffering from venereal disease, and only on women who become acknowledged prostitutes.

When we recognise these diseases as a menace to the public health, no matter in what way contracted, and proper instructions are given as to their prevention and transmission, a great step forward will have been taken toward lessening this evil. But it will require much skill in its management, and can only be brought about by the hearty coöperation of the medical profession. Does it not seem somewhat inconsistent to place a yellow card on the house in which there is a child with German measles, which is a self limited disease, trivial in its nature, and not pay the slightest attention to a disease which may be transmitted to a generation unborn?

A fond mother avoids with strictest care, all places where contagious diseases are known to exist, only to be rightly seized with horror upon the accidental discovery that one of her neighbors who has been fondling her child is suffering from syphilis, yet under present conditions there is no protection from this evil. What more can be done? It is the unanimous opinion of men and women, who have given this matter the most careful study and attention, that it lies in the early moral training and education in regard to sex matters of our children. This opinion is shared alike by clergymen, physicians, teachers and social workers. The real question is who shall be the teachers? The great responsibility of character building rests upon the parent in the home. The present tendency of so many to shirk that responsibility, is responsible for the great increase in all moral and social ills. A condition of confidence and trust must exist between parent and chlld. One of the first principles to be taught him, is to respect the rights of all living creatures; that the ability to torture and kill does not constitute the right to do so; that one of the chief duties of the stronger is to protect the weaker. With these principles firmly fixed in the mind of the three or four year old child, the corner stone of character and chivalry has been laid. Upon this foundation, truthfulness must be taught. In the mind of the child, truth clothed with common sense and tact far outweighs for moral character the most picturesque fairy tales which are designed to deceive and hide a truth which must come out later. It also tends to maintain a condition of confidence and respect, which the parent must possess if he expects to be trusted as a guide through the pitfalls, which are sure to come.

Character is the habit of right living. It is formed not taught; and the parent, who neglects his or her duty in this most impressionable age, forfeits an opportunity never to be regained. The parent, and not the chance and questionable acquaintance of the street, should teach the child in matters of sex; teach him the hygiene, not of certain parts of the body, but the whole body, and the habit of self control, and that reason and not passion should control their actions. Warn them against the promiscuous influences of quack newspaper advertisements, and teach the boys to consult the family physician when certain physiological questions trouble them. Teachers must be trained to assist and when necessary substitute, the parent in this instruction. It is generally believed that early instruction on the sex of plant life and primary zoological studies, will lead logically to a better understanding of sex matters. Gymnasium instructors can do much by personal contact and instruction. The clergyman, while teaching young men that their sins may be forgiven, should explain that the contagiousness of venereal disease is not quickly eradicated.

In what manner can we as physicians assist in bettering the present conditions? By explaining to our patients the seriousness of the crime of criminal abortion; by advising parents how best to teach their children sex hygiene; by explaining to young men the seriousness of venereal disease and its modes of transmissions, and insisting that they do not marry until cured; by the formation of societies for moral and social purity, to which the clergy, teachers and the parents are members; by insisting that the state health authorities recognises venereal diseases as a menace to public health and require these to issue slips of printed instructions in regard to the care and methods of transmission of venereal disease, which should be sent to all physicians with the request, that they be given to every patient who consults him for these diseases; by urging local Boards of Health to file records of cases of venereal disease which should be reported to them, without name or address, in order that more reliable data may be obtained upon which to formulate better plans of management.

We cannot hope to make rapid progress. We gather the fruits of the generation that precedes. Too many live with little thought of the next generation, forgetful of the fact that we form but a small link between the countless millions who have preceded us and the countless millions to come. Through our bodies must be transmitted what there is of the human life, be it good or bad. Of the woman who said "women are fools to have children," Woods Hutchinson has said "Neither the emancipated woman at the one end nor the prostitute at the other propagates her kind; for which society has reason to be thankful to both. Mrs. Philip Snowden a few days ago said "Women rule the world because they rule the child." I would substitute this wording—women would rule the world if they would train the child. For the nobleness of character of so many men and women in the world we owe them much, but much is neutralised by the thoughtlessness of others.

To the women of today is entrusted the nation of tomorrow. Then let us frankly acknowledge the supreme position of the faithful and devoted mother, and consider it an honor to assist her in her task.

709 South Warren Street.

